# Modeling a Dielectrophoretic Microfluidic Device with Vertical Interdigitated Transducer Electrodes for Separation of Microparticles Based on Size

**DOI:** 10.3390/mi11060563

**Published:** 2020-05-31

**Authors:** Fadi Alnaimat, Bobby Mathew, Ali Hilal-Alnaqbi

**Affiliations:** 1 Mechanical Engineering Department, United Arab Emirates University, Al Ain P. O. Box 15551, UAE; falnaimat@uaeu.ac.ae (F.A.); bmathew@uaeu.ac.ae (B.M.); 2 Abu Dhabi Polytechnic, MBZ campus, Abu Dhabi P. O. Box. 111499, UAE

**Keywords:** Interdigitated transducer electrodes, dielectrophoresis, microfluidics, microchannel, separation

## Abstract

This article conceptualizes and mathematically models a dielectrophoretic microfluidic device with two sets of interdigitated transducer vertical electrodes for separation of a binary heterogeneous mixture of particles based on size; each set of electrodes is located on the sidewalls and independently controllable. To achieve separation in the proposed microfluidic device, the small microparticles are subjected to positive dielectrophoresis and the big microparticles do not experience dielectrophoresis. The mathematical model consists of equations describing the motion of each microparticle, fluid flow profile, and electric voltage and field profiles, and they are solved numerically. The equations of motion take into account the influence of phenomena, such as inertia, drag, dielectrophoresis, gravity, and buoyancy. The model is used for a parametric study to understand the influence of parameters on the performance of the microfluidic device. The parameters studied include applied electric voltages, electrode dimensions, volumetric flow rate, and number of electrodes. The separation efficiency of the big and small microparticles is found to be independent of and dependent on all parameters, respectively. On the other hand, the separation purity of the big and small microparticles is found to be dependent on and independent of all parameters, respectively. The mathematical model is useful in designing the proposed microfluidic device with the desired level of separation efficiency and separation purity.

## 1. Introduction

**Figure 1:**
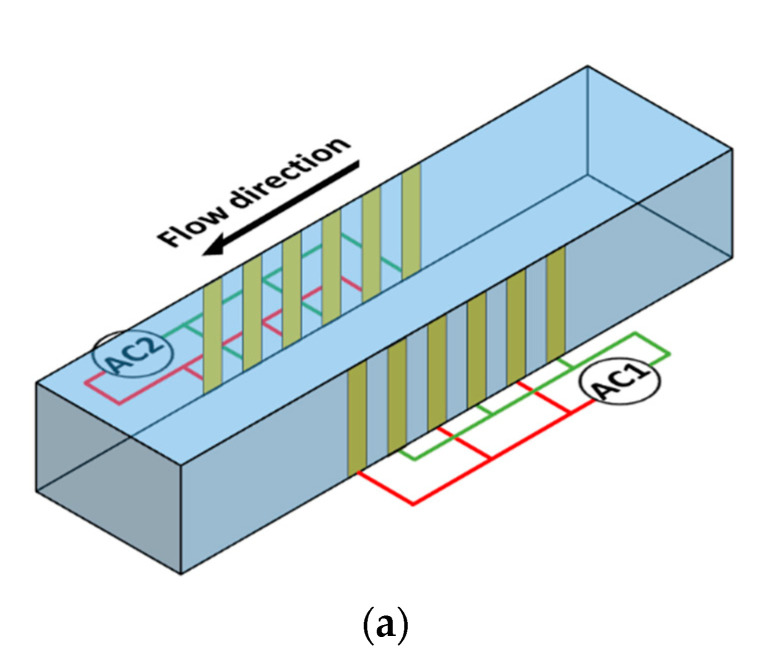

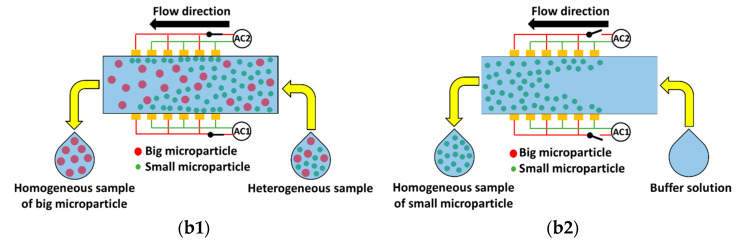
Schematic of the (**a**) proposed microfluidic device (perspective view) and (b) working of the device; (**b1**) capture of small microparticles on electrodes and collection of big microparticles at device exit during sample processing (top view) and (**b2**) collection of small microparticles at device exit after their release from electrodes while flushing device with buffer solution (top view).

Devices employing flow passages with hydraulic diameters smaller than 1 mm are referred to as microfluidic devices [1]. There are several advantages in employing microfluidic devices; these include low sample and reagent requirement, low power consumption, small footprint, and portability. One of the applications for which microfluidic devices are commonly employed is the separation of a heterogeneous sample into multiple homogeneous samples [2,3]; the basis of separation can be either size or type. Separation of a binary heterogeneous mixture into two homogeneous samples based on size requires every microparticle to be acted upon by an actuation force. There are several options for actuation forces that can be employed in microfluidic devices. One of the most commonly used actuation forces is that associated with dielectrophoresis (DEP) [4,5,6,7,8]. DEP is the phenomenon where in dielectric, but polarizable, microparticles suspended in a dielectric medium undergo translation when subjected to an electric field; the electric field needs to be non-uniform for DEP to exist [4,5,6,7,8]. DEP is specifically termed as positive DEP (pDEP) and negative DEP (nDEP) when the translation of the microparticles is towards the highest and lowest gradient of the electric field, respectively [4,5,6,7,8]. The force associated with DEP depends on several factors such as radius of the microparticles, permittivity of the medium, Clausius–Mossotti factor (*f*_CM_), and the magnitude and degree of non-uniformity of the electric field [4,5,6,7,8]. The *f*_CM_ is dependent on the permittivity and conductivity of the microparticle and medium as well as the operating frequency of the electric signal; microparticles experience pDEP and nDEP when real part of Clausius–Mossotti factor, *Re*(*f*_CM_), is positive and negative, respectively. The magnitude and degree of non-uniformity of the electric field depends on the dimensions and shape of the microchannel and the electrodes as well as the electrode configuration. Researchers have proposed electrode configurations for purposes of separation of microparticles based on size [4,5,6,7,8]. This article proposes a microfluidic device (Figure 1) for the separation of microparticles based on size using DEP. The device has one inlet and one outlet and consists of multiple interdigitated transducer (IDT) electrodes located on either sides of the microchannel. The set of electrodes on each side is independently controllable. In this device, separation is achieved by subjecting microparticles of a specific size to pDEP while keeping the microparticles of the alternative size unaffected. The microparticles that are subjected to pDEP will be attracted and captured on the electrodes while the microparticles that are not influenced by DEP will pass through the region of the electrodes unaffected and this leads to separation of microparticles based on size. The microparticles that are unaffected by DEP will be collected at the outlet as the sample is being processed, Figure 1b1; however, the microparticles captured by the electrodes will be collected from the same outlet, by switching off the electric power and flushing the microfluidic device with buffer solution, once the entire sample is processed Figure 1b2. The proposed device has the merit that it can handle high throughput in comparison with most devices proposed in literature [4,5,6,7,8]; this is primarily because the electric field does not decay along the height of the device. The proposed device also has the merit that it does not require focusing prior to separation of the heterogeneous sample. Three-dimensional microfabrication techniques required for realizing the conceptualized device are becoming common as can be observed from several articles in literature [5,9,10,11,12].

**Figure 2:**
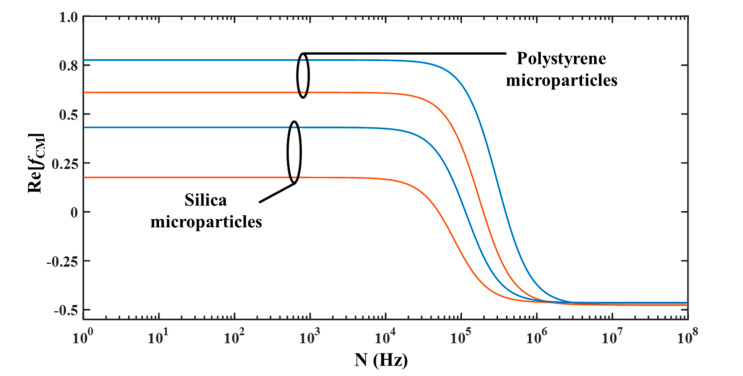
Variation of *Re*(*f*_CM_) with operating frequency for 2.5 μm and 5 μm microparticles (■: 2.5 μm, ■ 5 μm and σ_m_ = 0.0001 S/m).

Among all the different parameters influencing DEP force, the polarity of *Re*(*f*_CM_) can be easily altered by varying the operating signal. Thus, the operating frequency of the proposed device should be such that microparticles of a particular size will experience pDEP while microparticles of other size will experience negligible DEP. Figure 2 shows the variation of *Re*(*f*_CM_) with operating frequency for polystyrene (*ε_ps_* = 2.55 and *K_s,ps_* = 2.85 nS) and silica microparticles (*ε_s_* = 3.8 and *K_s,s_* = 0.82 nS) [13]. It can be noticed that *Re*(*f*_CM_) is dependent on the operating frequency. It can be noticed that for both types of microparticles, irrespective of their radii, the *Re*(*f*_CM_) is positive and negative at low and high operating frequencies, respectively. Furthermore, it can be noticed from Figure 2 that there exists a unique operating frequency, for a mixture of two different sized microparticles, at which the small microparticle experiences pDEP while the big microparticle experiences zero DEP and the proposed device should be operated at this frequency for achieving separation based on size. Frequency at which a microparticle experiences zero DEP force is called cross-over frequency; thus, the proposed device needs to be operated at the cross-over frequency of the big microparticle. Figure 2 has been developed using Equations (1) and (2); cross-over frequency (*N_cr_*) of a microparticle can be calculated using Equation (3).

(1) $$Re\left[f_{CM}\right]=\frac{\left(\varepsilon_{e}+2\varepsilon_{m}\right)\left(\varepsilon_{e}-\varepsilon_{m}\right)+\frac{\left(\sigma_{e}+2\sigma_{m}\right)\left(\sigma_{e}-\sigma_{m}\right)}{\omega^{2}}}{{\left(\varepsilon_{e}+2\varepsilon_{m}\right)}^{2}+\frac{{\left(\sigma_{e}+2\sigma_{m}\right)}^{2}}{\omega^{2}}}$$

(2) $$\sigma_{e}=\sigma_{bulk,e}+2\frac{K_{s,e}}{r_{e}}$$

(3) $$N_{cr}=\frac{\omega }{2\pi }=\frac{1}{2\pi }\sqrt{\frac{\left(\sigma_{e}+2\sigma_{m}\right)\left(\sigma_{m}-\sigma_{e}\right)}{\left(\varepsilon_{e}+2\varepsilon_{m}\right)\left(\varepsilon_{e}-\varepsilon_{m}\right)}}$$

çetin et al. [14] modeled and constructed a DEP-based microfluidic device for separation of microparticles based on size. The device employs a pair of opposing vertical electrodes; one of the electrodes is finite sized while the other electrode is very long in comparison. The mixture with different sized microparticles are focused near the sidewall with the small electrode using sheath flow. In the vicinity of the small electrode, each microparticle is subjected to nDEP force and as it is proportional to the size of the microparticle, the bigger microparticles are pushed further into the microchannel than smaller microparticles. This splits the mixture into two samples with each having microparticles of a particular size. çetin et al. [14] modeled the trajectory of microparticles in the device; the model consisted of Stokes equation, the equation of electric potential, and equations of motion that considered the influence of forces such as inertia, drag, and DEP. The working of the device is demonstrated by separation a mixture of 5 and 10 μm latex microparticles into homogeneous samples of 5 and 10 μm [14]. Wang et al. [15] constructed and tested a DEP-based microfluidic device, with one set of vertical IDT electrodes located on each of the sidewalls, for separation based on type. The voltage and frequency of operation of one set of IDT electrodes is different from the other. In one instance, this caused one set of IDT electrodes to simultaneously subject the first type of microparticle to weak nDEP and the second type of microparticle to strong nDEP while the other set of IDT electrodes simultaneously subjected first and second types of microparticles to strong nDEP and weak nDEP, respectively. This difference in DEP forces experienced by microparticles allowed for separating a heterogeneous mixture of microparticles and cells into two homogenous samples based on type. çetin and Li [16] modeled a DEP-based microfluidic device for separation of microparticles based on size. The device consists of two vertical electrodes with one electrode placed upstream of a curved section, of the microchannel, while placing the second electrode downstream of the same. All big microparticles are subjected to pDEP which causes them to move towards the inner wall of the curved section while the small microparticles are subjected to nDEP which causes them to move towards the outer wall of the same thereby leading to desired separation based on size. Kang et al. [17] developed a microfluidic DEP-based device for the separation of microparticles. The device uses a pair of vertical electrodes with one electrode placed, on one of the sidewalls, upstream and the other electrode placed, on the opposing sidewall, downstream of a constriction in the microchannel; moreover, the constriction is closer to the upstream electrode. The incoming microparticles are focused using sheath flow prior to reaching the constriction and as the microparticles pass through the constriction, they are pushed against one of its sidewalls by nDEP force. Subsequently, streamlines passing through the center of the microparticle carry them out of the constriction and as these streamlines are different, the desired separation based on size is achieved. Faraghat et al. [18] developed a DEP-based filter for type-based separation of cells. The filter consists of multiple layers of electrode sandwiched between insulating layers through which several through holes are realized. An electric field is set up between two neighboring electrode layers. With this device, it is possible to subject entities to either pDEP or nDEP; entities subjected to pDEP are attracted and captured on the walls of the through holes while those entities experiencing nDEP are focused at the center of the through holes thereby achieving he desired separation. Mathew et al. [19,20,21] and Alazzam et al. [22] modeled several microfluidic devices employing spatially varying electric field for realizing field flow fractionation to achieve type based separation of microparticles. In this device the microparticles are subjected to nDEP and sedimentation forces in the vertical direction and this leads to levitation of the microparticles. The levitation height is dependent on the permittivity and density of the microparticle thereby allowing for separation of microparticles. Mathew et al. [19] employed multiple finite sized IDT electrodes located on the bottom surface of the microchannel while Mathew et al. [20] conceptualized a device with multiple finite sized and continuous electrodes on the bottom and top surfaces, respectively. The microfluidic device of Mathew et al. [21] and Alazzam et al. [22] consisted of multiple finite sized electrodes located on its top and bottom surfaces; in Mathew et al. [21], the electrodes on both surfaces are aligned while in Alazzam et al. [22], the electrodes on one surface is aligned with the electrode gap on the opposite surface. Alnaimat et al. [13] developed the mathematical model of a microfluidic device, employing multiple finite sized planar IDT electrodes located on the bottom surface, for separation of microparticles based on type. In this device, one type of microparticle is subjected to pDEP while the other type of microparticle is subjected to nDEP; the microparticles experiencing pDEP are attracted and captured on the electrodes while the microparticles subjected to nDEP are levitated above the electrodes and this achieves separation based on type.

The microfluidic device conceptualized in this document can find application in the area of disease diagnosis, especially that requiring investigation of blood [23,24]. Diagnosis of certain illness depends on identifying foreign entities or rare cells in blood samples [23,24]. For these illnesses, the proposed microfluidic device can be used for identifying foreign entities or rare cells as long as there sizes are different from that of regular cells.

This work is the first to model the microfluidic device shown in Figure 1 while working under the proposed scheme with the aim of separation of microparticles based on size. The model takes into account the influence of all forces associated with the movement of microparticles in microfluidic devices; the forces include that associated with inertia, drag, gravity, buoyancy, and DEP. The inclusion of forces associated with inertia and drag allows for determining the time and length required for achieving a desired performance metric in the proposed device.

## 2. Mathematical Modeling

The mathematical model of the microfluidic device conceptualized in the previous section of this document is detailed in this section. The mathematical model of the microfluidic device consists of equations of motion, equation of fluid flow, equations of electric voltage, and field. In microfluidic devices the flow is very small and one-dimensional, i.e., flow has velocity only in axial direction. Equation (4) represents the equations of the motion while Equation (5) is the equation of fluid flow (one dimensional). Equations (6) and (7) describe the electric voltage and electric field inside the microchannel, respectively.

(4) $$m_{e}\frac{d}{dt^{2}}\left(x_{e}i+y_{e}j+z_{e}k\right)=\sum \left(F_{e,\bar{x}}i+F_{e,\bar{y}}j+F_{e,\bar{z}}k\right)$$

(5) $$\left(\frac{\partial^{2}}{\partial {\bar{y}}^{2}}+\frac{\partial^{2}}{\partial {\bar{z}}^{2}}\right)u_{m,\bar{x}}=\frac{1}{\mu_{m}}\frac{d}{d\bar{x}}P\;$$

(6) $$\left(\frac{\partial^{2}}{\partial {\bar{x}}^{2}}+\frac{\partial^{2}}{\partial {\bar{y}}^{2}}+\frac{\partial^{2}}{\partial {\bar{z}}^{2}}\right)V_{RMS}=0$$

(7) $$E_{\bar{x}}i+E_{\bar{y}}j+E_{\bar{z}}k=-\left(\frac{\partial }{\partial \bar{x}}i+\frac{\partial }{\partial \bar{y}}j+\frac{\partial }{\partial \bar{z}}k\right)V_{RMS}$$

The equation of motion, Equation (4), accounts for forces such as that associated with gravity, drag, buoyancy, and DEP. The force associated with gravity and buoyancy are provided in Equations (8) and (9), respectively. The force associated with gravity and buoyancy have only one component and it is in the vertical direction. The force related to drag that is acting on the microparticle is shown in Equation (10) while that related to DEP is presented in Equation (11). The solution of equation of fluid flow, Equation (5), is required for determining drag which in turn is required for calculating the trajectory of the microparticles and is provided in Equation (12) [7].

(8) $$F_{g,\bar{x}}i+F_{g,\bar{y}}j+F_{g,\bar{z}}k=-\frac{4}{3}\pi r_{e}^{3}\rho_{e}gk$$

(9) $$F_{b,\bar{x}}i+F_{b,\bar{y}}j+F_{b,\bar{y}}k=\frac{4}{3}\pi r_{e}^{3}\rho_{m}gk$$

(10) $$F_{drag,\bar{z}}i+F_{drag,\bar{y}}j+F_{drag,\bar{z}}k=6\pi \mu_{m}r_{e}\left[\left({u_{m}|}_{X_{e}}-\frac{d}{dt}x_{e}\right)i-\frac{d}{dt}y_{e}j-\frac{d}{dt}z_{e}k\right]$$

(11) $$F_{DEP,\bar{x}}i+F_{DEP,\bar{y}}j+F_{DEP,\bar{z}}k=2\pi \varepsilon_{m}\varepsilon_{0}r_{e}^{3}Re\left[f_{CM}\right]\left({\frac{\partial E_{RMS}^{2}}{\partial \bar{x}}|}_{X_{e}}i+{\frac{\partial E_{RMS}^{2}}{\partial \bar{y}}|}_{X_{e}}j+{\frac{\partial E_{RMS}^{2}}{\partial \bar{z}}|}_{X_{e}}k\right)$$

(12) $${u_{m,\bar{x}}|}_{X_{e}}=\frac{48Q_{m}\overset{\infty }{\underset{i=1,3,5}{\sum }}\left(\frac{{\left(-1\right)}^{\left(\frac{i-1}{2}\right)}}{i^{3}}\right)\left\{1-\frac{\cosh\left[i\frac{\pi }{W_{ch}}\left(\frac{H_{ch}}{2}-z_{e}\right)\right]}{\cosh\left(\frac{i\pi }{2}\frac{H_{ch}}{W_{ch}}\right)}\right\}\cos\left[i\frac{\pi }{W_{ch}}\left(\frac{W_{ch}}{2}-y_{e}\right)\right]}{\pi^{3}W_{ch}H_{ch}\left[1-\frac{192\;W_{ch}}{\pi^{5}H_{ch}}\overset{\infty }{\underset{i=1,3,5}{\sum }}\frac{\tanh\left(i\frac{\pi }{2}\frac{H_{ch}}{W_{ch}}\right)}{i^{5}}\right]}$$

Equation (4) is solved using Finite Difference Method (FDM). For this, the differential terms are replaced by difference terms. The second order differential terms are replaced by second order central difference term. The time step is maintained at 10^−5^ s. A MATLAB program was developed for solving Equation (4). For solving Equation (4), there are two initial conditions as depicted in Equation (13) and Equation (14). The initial displacements of the microparticle are presented in Equation (13) while the initial velocities of the microparticle are shown in Equation (14). The microparticle can start from any location across from the cross-section of the microchannel and this represents the initial displacement of the microchannel. The initial velocities of the microparticle are same as that of the fluid at the initial location of the microparticle.

(13) $${\left[{x_{e}|}^{t=0},{y_{e}|}^{t=0},{z_{e}|}^{t=0}\right]}^{T}={\left[0,W_{0},H_{0}\right]}^{T}$$

(14) $$\frac{d}{dt}{\left[{x_{e}|}^{t=0},{y_{e}|}^{t=0},{z_{e}|}^{t=0}\right]}^{T}={\left[{u_{m,\bar{x}}|}_{X_{e}},0,0\right]}^{T}$$

**Figure 3:**
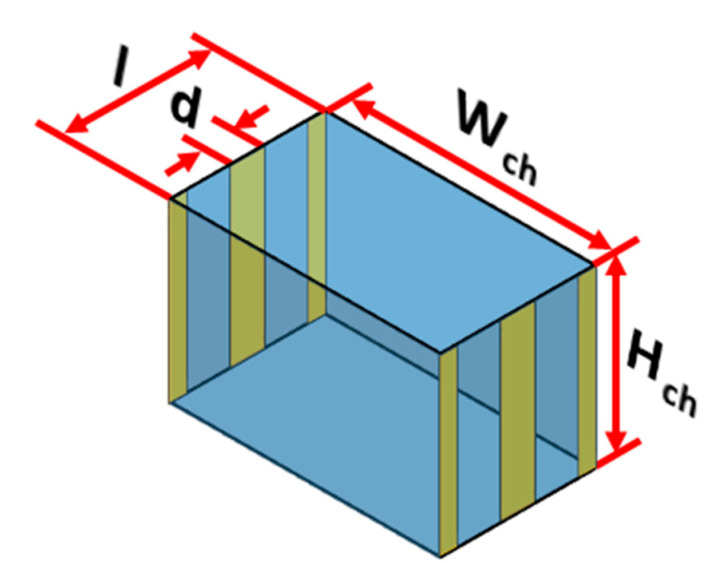
Schematic of the repeating unit of the proposed microfluidic device.

The electric field required for solving Equation (4) is obtained by solving Equation (7). The electric field depends on the electric potential and thus it needs to be determined throughout the microchannel and for this Equation (6) needs to be solved. Equation (6) is solved using FDM as well after replacing second order differential terms by second order central difference schemes; the boundary conditions associated with Equation (6) include known voltages on the electrodes while the remaining boundaries are assumed to be insulated. It needs to be stressed here that solving Equation (6) for the entire microchannel will be computationally taxing and time consuming. To overcome this, Eqution (6) is solved only in a repeating unit of the microchannel and later information on electric voltage inside the repeating unit is mapped on the entire microchannel; similar apporach is taken with regards to the electric field and DEP force. The repeating unit is schematically shown in Figure 3; each repeating unit contains one electrode pair on either side of the microchannel.The internode distance for implementing FDM is maintained at 1 μm. The several linear equations generated by application of FDM are solved using Gauss-Seidel method. Electric field is calculated after replacing the first order differential terms of Equation (7) by second order forward/central/backward difference schemes. A MATLAB program was developed for solving Equation (6).

The performance of microfluidic devices employed for separation is quantified in terms of separation efficiency (SE), Equation (15), and separation purity (SP), Equation (16) [13]. SE and SP are quantified using the position of the microparticles at the exit of the microchannel. SE is the ratio of the number of microparticles of a particular size at the outlet to the number of microparticles of the same size at the inlet. SE represents the percentage of the total number of microparticles of a particular size separated using the device compared with the number of microparticles of the same size in the heterogeneous sample. SP is the ratio of the number of microparticles of a particular size at the outlet of the device to the total number of microparticles at the outlet.

(15) $$SE\left(A\right)=\frac{\#\;of\;microparticles\;of\;size\;\prime A\prime \;at\;outlet}{\#\;of\;microparticles\;of\;size\;\prime A\prime \;at\;inlet}$$

(16) $$SP\left(A\right)=\frac{\#\;of\;microparticles\;of\;size\;\prime A\prime \;at\;outlet}{\#\;of\;microparticles\;of\;all\;sizes\;at\;outlet}$$

All studies are done by uniformly releasing several microparticles from the inlet of the microchannel and subsequently tracking their trajectories to determine *SE* and *SP*; this approach has been previously adopted by researchers [13,25,26]. One of the assumptions of this model is that the microparticles do not experience Brownian motion. This assumption is acceptable as long as the microparticles are greater than 1 μm as established in literature [27,28,29]. Additionally, it is assumed that there is no particle-to-particle interaction inside the microchannel and this is a reasonable assumption as long as the sample handled in the microfluidic device is dilute and it is often the case when employing microfluidic devices [30].

## 3. Results and Discussions

**Figure 4:**
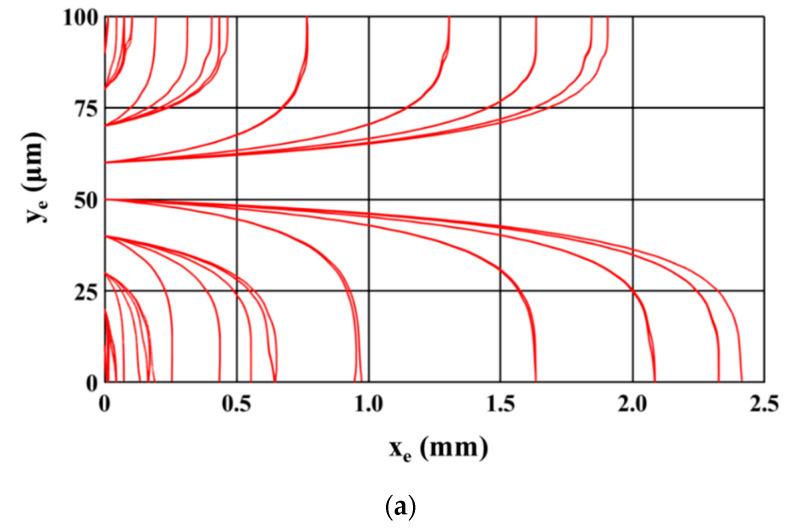

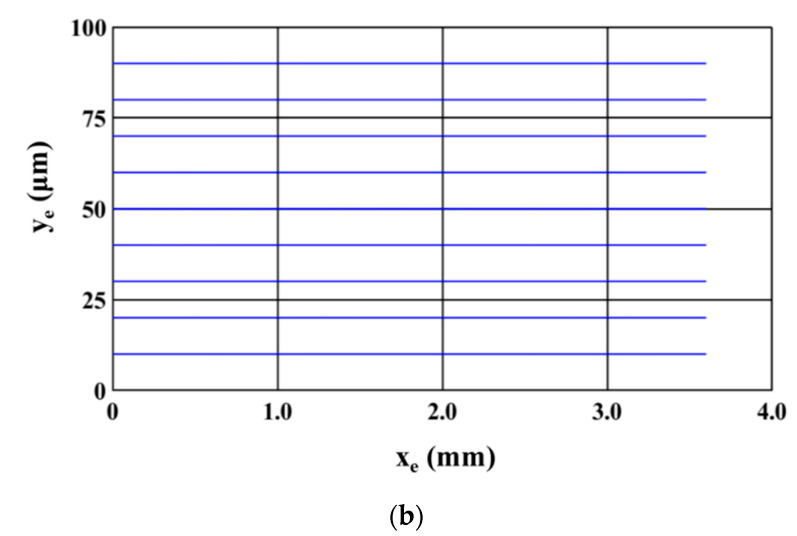
Top view of trajectory of microparticles (**a**) 2.5 μm and (**b**) 5 μm (*V_1_* = 20 V_pp_, *V_2_* = 12.5 V_pp_, *Q_m_* = 200 μL/h, *l* = 120 μm, *d* = 30 μm, *n* = 30, *N* = *N_cr,5_* = 192.8 kHz).

Figure 4 shows the trajectory of the microparticles as the sample is being processed inside the microfluidic device. The sample introduced at the inlet of the microchannel consists of equal numbers of 2.5 μm (radius) and 5 μm (radius) polystyrene microparticles. Figure 4a,b show the path of 2.5 μm and 5 μm microparticles; two different figures are used so that the paths of each size of microparticles are clearly visible. For this study, microparticles of a particular size are uniformly released from the inlet of the microchannel; microparticles are released from 81 locations across the inlet of the microchannel. It can be noticed that all 2.5 μm microparticles are attracted to and captured on the electrodes while the 5 μm polystyrene microparticles travel through the microchannel unaffected. This depicts the ability of the microfluidic device to achieve separation based on size. The following parts of this section details the study carried out to understand the influence of operating and geometric parameters on SE and SP. The operating and geometric parameters considered include applied voltage, electrode dimensions, volumetric flow rate, and number of electrodes.

**Figure 5:**
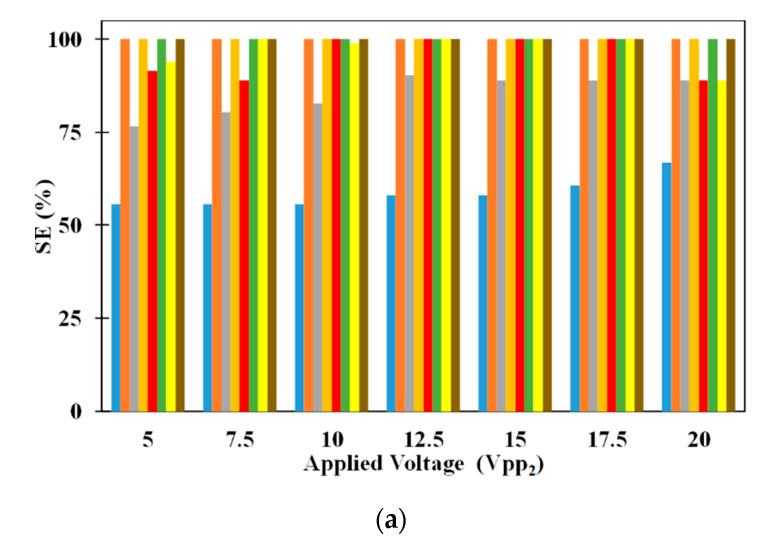

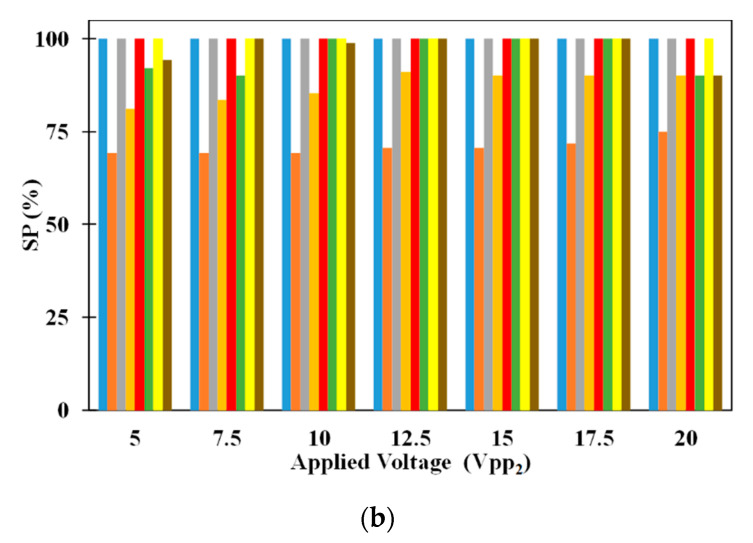
Influence of electrode dimensions on (**a**) SE and (**b**) SP for *l* = 40 μm and *d* = 10 μm (■: 2.5 μm, ■ 5 μm), *l* = 80 μm and *d* = 20 μm (■: 2.5 μm, ■ 5 μm), *l* = 120 μm and *d* = 30 μm (■: 2.5 μm, ■ 5 μm), and *l* = 160 μm and *d* = 40 μm (■: 2.5 μm, ■ 5 μm); *n* = 30, *Q_m_* = 200 μL/h, *W_ch_* = 100 μm, *H_ch_* = 100 μm, *V_pp1_* = 20 V_pp_, *N* = *N_cr,5_* = 192.8 kHz.

Figure 5 shows the influence of electrode dimensions on SE and SP. It can be noticed that SE of 5 μm microparticle is independent of electrode dimensions for all applied electric voltages. While inside the microchannel, the 5 μm microparticles do not experience DEP as it needs to just remain suspended in the medium thereby making the SE of the same independent of the dimensions of the electrodes. On the other hand, SE of 2.5 μm microparticles depends on the electrode dimensions irrespective of the applied voltages (except for certain electrode dimensions operating at V_pp1_ = V_pp2_). With the increase in electrode dimensions, the SE of 2.5 μm microparticles increases for all applied electric voltages. The residence time, of 2.5 μm microparticles, increases with the increase in electrode dimensions and this leads to a greater number of microparticles being captured on the electrodes thereby leading to the observed increase in SE, of 2.5 μm microparticles, for a specific applied electric voltage; residence time is the time spent by a microparticle inside the microchannel. For certain cases of equal applied electric voltages, all 2.5 μm microparticles released from the center of the inlet of the microchannel experience same pDEP from both sets of IDT electrodes due to which they remain uncaptured. This coupled with the fact that the number of 2.5 μm microparticle captured and subsequently released remain the same, the SP of the same remains independent of the electrode dimensions.

The SP of both 2.5 μm and 5 μm microparticles are shown in Figure 5a as well. It can be noticed that changes in electrode dimensions do not influence the SP of 2.5 μm microparticles for all applied electric voltages. SP of 2.5 μm microparticles is calculated only using the number of 2.5 μm microparticles captured on the electrodes and as these are released for collection after processing of the sample, they appear at the exit without the presence of 5 μm microparticles thereby achieving SP of 100%. On the other hand, SP of 5 μm microparticles is influenced by electrode width and this is related to the number of 2.5 μm microparticles captured on the electrodes. As the number of the 2.5 μm microparticles captured on the electrodes increase due to increase in applied electric voltage or electrode width, the number of 2.5 μm polystyrene microparticles collected along with 5 μm microparticles decreases thereby leading to increase in the SP of the same.

**Figure 6:**
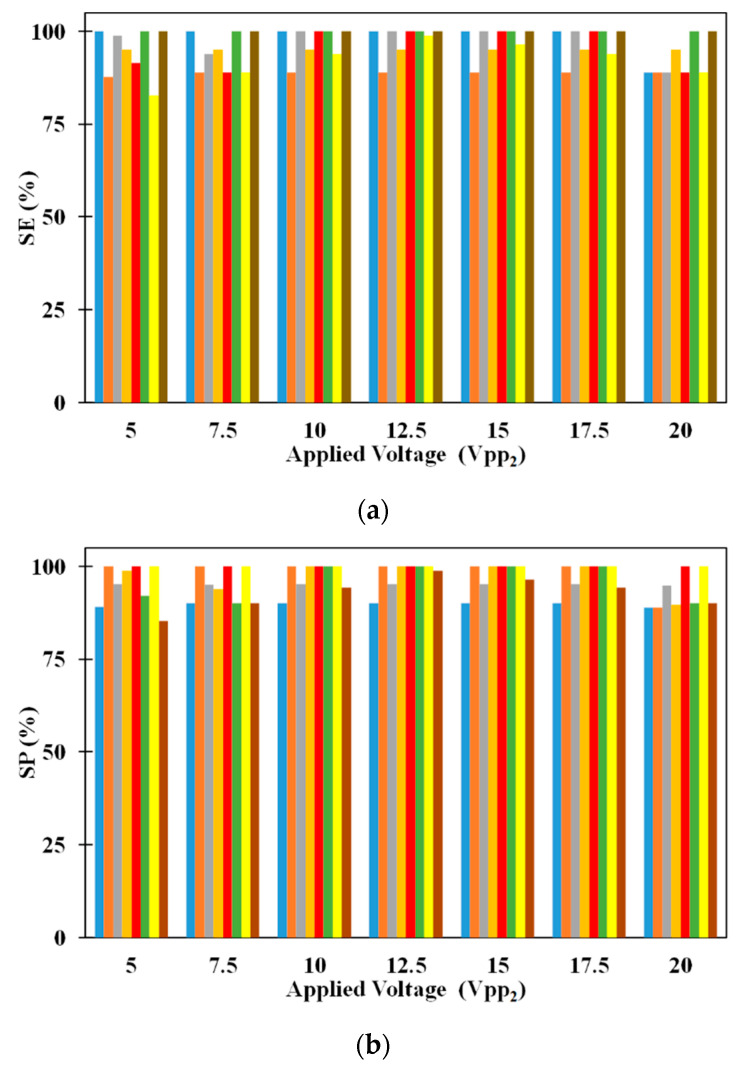
Influence of volumetric flow rate on (**a**) SE and (**b**) SP for *Q_m_* = 50 μL/h (■: 2.5 μm, ■ 5 μm), *Q_m_ =* 100 μL/h (■: 2.5 μm, ■ 5 μm), *Q_m_ =* 200 μL/h (■: 2.5 μm, ■ 5 μm), and *Q_m_ =* 300 μL/h (■: 2.5 μm, ■ 5 μm); *n* = 30, *W_ch_* = 100 μm, *H_ch_* = 100 μm, *V_pp1_* = 20 V_pp_, *N* = *N_cr,5_* = 192.8 kHz.

Figure 6a provides the comparison between SE of 2.5 μm and 5 μm microparticles for different volumetric flow rates. It can be noticed that increase in volumetric flow rate decreases the SE of 2.5 μm when all other parameters are held constant. On the other hand, changes in volumetric flow rate, irrespective of the other parameters, does not influence SE of 5 μm microparticle. With the increase in volumetric flow rate, the residence time of all microparticles in the microchannel decreases. This reduction in residence time reduces the number of 2.5 μm microparticles captured on the electrodes which in turn negatively affects the SE of the same. Regarding the SE of 5 μm microparticle, it is below 100% for low volumetric flow rates; the increased residence time, associated with low volumetric flow rates, leads to the fall of several 5 μm microparticles to the bottom surface of the microchannel thereby preventing all of them from reaching the exit of the microchannel. At high volumetric flow rates, the residence time is low to prevent the fall of any 5 μm microparticles to the bottom surface of the microchannel thereby allowing all to remain suspended in the medium and subsequently reach the exit of the microchannel. Also, it can be noticed that increase in applied voltage increases the SE of 2.5 μm microparticles for a particular flow rate. Increase in applied voltage increases the pDEP force acting on the 2.5 μm microparticles and this leads to the observed increase in capture of the same with the increase in applied electric voltages. Additionally, it can be observed that the increase in applied electric voltages does not affect the SE of 5 μm microparticle since the separation of these microparticles is not dependent on them being captured on the electrodes. The SE of 2.5 μm microparticle is independent of the volumetric flow rate at applied electric voltages of 20 V_pp_; this is because the microparticles in the vertical plane through the center the microchannel experience equal pDEP from both sets of electrodes thereby preventing them from being attracted to the electrodes and subsequently captured on the same.

The effect of volumetric flow rate on SP of 2.5 μm and 5 μm microparticles are shown in Figure 6b. The SP of 2.5 μm microparticle is influenced by the volumetric flow rate at low and high volumetric flow rates, respectively. At low volumetric flow rates, several 5 μm microparticles drop to the bottom surface of the microchannel and these are collected along with the 2.5 μm microparticles captured on the electrodes thereby causing the SP of 2.5 μm microparticles to be dependent on volumetric flow rate. On the other hand, no 5 μm microparticles drop to the bottom surface of the microchannel at high volumetric flow rates and this maintains the SP of 2.5 μm microparticles at 100%. Regarding the SP of 5 μm microparticles, they are influenced by volumetric flow rate. With the increase in volumetric flow rate, for a specific applied electric voltage, the number of 2.5 μm microparticles captured on the electrodes decrease. The uncaptured 2.5 μm microparticles appear at the exit of the microchannel along with 5 μm microparticles thereby affecting the SP of 5 μm microparticles. Applied electric voltage also influences the SP of 5 μm microparticles; an increase in applied electric voltage increases the SP of 5 μm microparticles. This is because with the increase in applied electric voltage the number of 2.5 μm microparticles captured on the electrodes increase there by reducing the number of 2.5 μm microparticles appearing at the exit along with 5 μm microparticles and subsequently enhancing SP of 5 μm microparticles.

**Figure 7:**
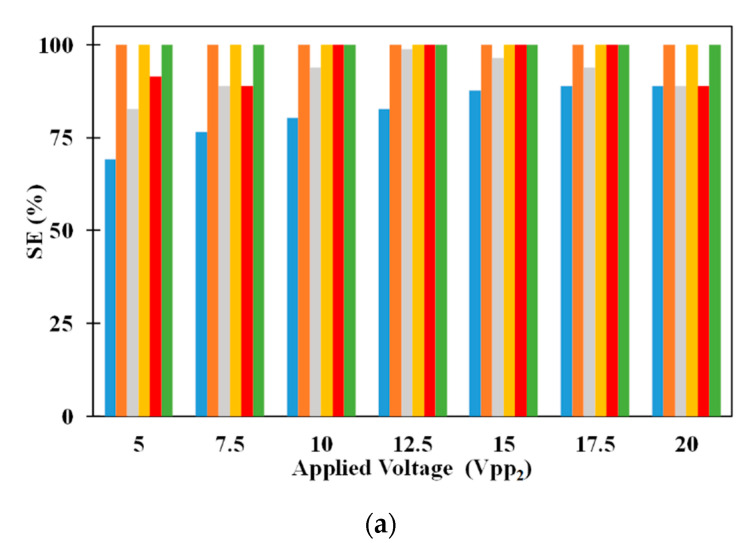

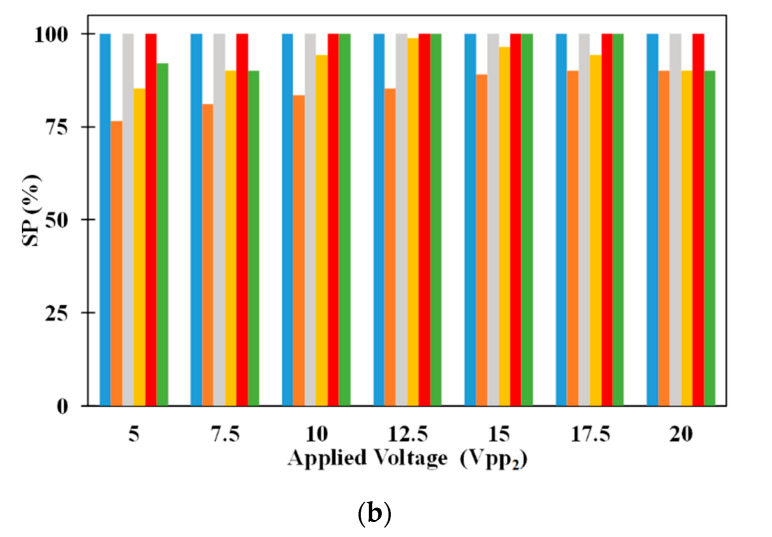
Influence of number of electrodes on (**a**) SE and (**b**) SP for *n* = 10 (■: 2.5 μm, ■ 5 μm), *n* = 20 (■: 2.5 μm, ■ 5 μm), and *n* = 30 (■: 2.5 μm, ■ 5 μm); *Q_m_* = 200 μL/h, *W_ch_* = 100 μm, *H_ch_* = 100 μm, *V_pp1_* = 20 V_pp_, *N* = *N_cr,5_* = 192.8 kHz.

The influence of number of electrodes on the SE of 2.5 μm and 5 μm microparticles is provided in Figure 7a. It can be noticed that increase in the number of electrodes increases the SE of 2.5 μm microparticle irrespective of the applied electric voltages. With the increase in the number of electrodes, the length of the microchannel increases which increases the residence time of the microparticles thereby leading to increase in the SE of 2.5 μm microparticles. It can also be noticed that an increase in applied electric voltage, for a specific number of electrodes, increases the SE of 2.5 μm microparticle. With the increase in applied electric voltages, the pDEP forces attracting the microparticles to the electrodes increase and this is the reason for the observed increase in SE of 2.5 μm microparticle. SE of 2.5 μm microparticle is independent of the number of electrodes when the applied voltages are 20 V_pp_. At 20 V_pp_, the microparticles in the vertical plane passing through the center of the microchannel experience same pDEP force from both sets of electrodes due to which they remain uncaptured and subsequently the associated SE lower than 100%. At equal applied electric voltages of 20 V_pp_, the pDEP force is strong to capture all but those microparticles in the vertical plane through the center of the microchannel and thus, the SE is independent of the number of electrodes. On the other hand, the SE of 5 μm microparticles is independent of the number of electrodes; to achieve separation of the heterogeneous mixture, the 5 μm microparticles just need to remain suspended in the microchannel and this is unaffected by the number of electrodes thereby maintaining the SE of the constant at 100%.

Figure 7b also shows the variation of SP, of, 2.5 μm and 5 μm microparticles, with number of electrodes. It is very clear from Figure 7b that SP of 2.5 μm microparticles are independent of the number of electrodes and applied electric voltages. This is the result of the SE of 5 μm microparticle being independent of the number of electrodes. As SE of 5 μm microparticle is 100% for all cases, no 5 μm microparticles will be collected with the 2.5 μm microparticles thereby making the same 100%. On the other hand, the SP of 5 μm microparticle varies with number of electrodes and applied electric voltages. In several instances, all 2.5 μm microparticles are not captured on the electrodes due to which many are collected, at the outlet, along with 5 μm microparticles and this makes the SP of 5 μm microparticles dependent on the number of number of electrodes and voltages. For equal applied electric voltages of 20 V_pp_, the SP of 5 μm microparticle is constant irrespective of the number of electrodes. At equal electric voltages of 20 V_pp_, none of the 2.5 μm microparticles in the vertical plane passing through the center of the microchannel are captured on the electrodes and subsequently they appear at the exit along with the 5 μm microparticles. As the number of 2.5 μm microparticles appearing at the exit of microchannel, along with 5 μm microparticle, is always same, the SP of 5 μm remains constant.

## 4. Device Limitations

For both equal and unequal applied electric voltages, there exists a vertical plane in the microchannel in which the DEP force is zero. This implies that the small microparticles in this vertical plane will not be attracted to the electrodes thereby influencing the SE of small microparticle and the SP of the big microparticle. This is the limitation of the microfluidic device; nevertheless, the device still exhibits good SE and SP as can be seen from the previous section.

## 5. Conclusions

This document presents a dielectrophoretic microfluidic device with multiple finite sized vertical electrodes, arranged in interdigitated transducer configuration, for separation of microparticles based of size. The electrodes on one side of the wall are controlled independently from the electrodes on the other side of the wall. In this device, microparticles of a specific size are captured on the electrodes while bigger microparticles pass through the microchannel unaffected to realize separation. The influence of parameters (operating and geometric) on the performance metrics, separation efficiency and separation purity, are quantified in this document. Separation efficiency of the small microparticles increase with the increase in applied electric voltage, width of electrodes, and number of electrodes while it reduces with the increase in the volumetric flow rate; the variation in separation efficiency can be attributed to the residence of the microparticles in the device. The separation efficiency of the 5 μm microparticle is always 100% as separation does not depend on the dielectrophoretic force. The separation purity of the 2.5 μm microparticles is independent of the applied electric voltage, electrode dimensions and the number of electrodes; however, volumetric flow rate influences separation purity. Separation purity of 5 μm microparticles are influenced by applied electric voltages, width of electrodes, volumetric flow rate, and number of electrodes and it is observed to increase with the increase in these parameters; this improvement in separation purity of 5 μm microparticles is due to the increase in separation efficiency of 2.5 μm microparticles with the increase in the parameters.

## Nomenclature

*d*: width of repeating unit (m or μm)
*E*: electric field
*H_0_*: initial displacement along microchannel height (m or μm)
*H*: height (m or μm)
*g*: acceleration due to weight (m/s^2^)
*F*: force (N)
*K_s_*: surface conductance (S)
*L*: length of the electrode (m or μm)
*N*: number of microparticles for determining ∆W and ∆H (-)
*n*: number of electrodes (-)
*Q*: flow rate (m^3^/s or μL/h)
*Re*(*f*_CM_): Clausius–Mossotti factor (-)
*r*: radius (m or μm)
*SE*: separation efficiency
*SP*: separation purity
*t*: time (s)
*u*: velocity of medium (m/s)
*V*: voltage (V)
*W_0_*: initial displacement along microchannel width (m or μm)
*W*: width (m or μm)
***X***: displacement vector (m or μm)
*x*: displacement in the x-direction (m)
*y*: displacement in the y-direction (m)
*z*: displacement in the z-direction (m)
**Greek alphabet**
*ε*: permittivity (-)
*ε_o_*: permittivity of free space (F/m)
*σ*: conductivity (S/m)
*ρ*: density (kg/m^3^)
*ω*: operating frequency (rad/s)
**Subscripts**
*2.5*: 2.5 μm microparticle
*5*: 5 μm microparticle
*ch*: microchannel
*cr*: cross-over
*Drag*: drag
*DEP*: dielectrophoresis
*e*: entity
*RMS*: root mean square
*m*: medium
